# Chewing khat and risky sexual behavior among residents of Bahir Dar City administration, Northwest Ethiopia

**DOI:** 10.1186/s12991-018-0194-2

**Published:** 2018-06-12

**Authors:** Andargie Abate, Minale Tareke, Mulat Tirfie, Ayele Semachew, Desalegne Amare, Emiru Ayalew

**Affiliations:** College of Medicine and Health Science, Bahir Dar University, Bahir Dar, Ethiopia

**Keywords:** Khat chewing, Risky sexual behavior, Bahir Dar, Ethiopia

## Abstract

**Introduction:**

Khat is a well-known natural stimulant and is widely used in Ethiopia, particularly in Bahir Dar city. Khat chewing is linked with risky sexual behaviors.

**Objective:**

The study was aimed to determine the prevalence of chewing khat and its relation with risky sexual behaviors among residents of Bahir Dar City administration, Northwest Ethiopia.

**Methods:**

A community based cross-sectional study was conducted from January to February, 2016. The data were collected using an interviewer administered structured questionnaire. Logistic regression analysis was applied to assess association between dependent and explanatory variables.

**Results:**

The proportion of lifetime and current chewing khat among the study participants were 25.7 and 19.5%, respectively. Males (AOR 5.0; 95% CI 3.0–8.2) than females, merchants (AOR 4.9; 95% CI 2.6–9.3) than government employees, and those with average monthly income of ≥ 3001 Ethiopian birr (AOR 2.4; 95% CI 1.2–4.8) than ≤ 1000 had an increased current chewing khat prevalence. Having lifetime history of chewing khat was significantly associated with ever had sexual intercourse, having extra sexual partners, watching pornographic film and self-reported sexually transmitted infections.

**Conclusion:**

Chewing khat is associated with increment of having risky sexual behaviors and self-reported sexually transmitted infections. Harm reduction measures are needed to prevent the community from engaging in khat use and risky sexual behaviors.

## Introduction

Khat is a natural stimulant from the *Catha edulis* plant which is grown in southern Arabia and Eastern Africa, and primarily in the countries of Ethiopia, Somalia, Kenya and Yemen [1–3]. It is a bushy plant whose leaves are chewed for social and psychological reasons due to the active psycho-stimulant substance known as cathinone which affects central nervous system (CNS) like amphetamine. The leaves are freshly stripped from the trees at dawn and rapidly distributed in regions where Khat chewing is widespread since cathinone can be found only in fresh *Catha edulis* leaves [1, 2, 4].

An estimated 10 million people worldwide chew khat, and commonly found and consumed in the southwestern part of Arabian Peninsula and East Africa, and immigrant communities living in Europe and North America [3, 5, 6]. Similarly, Ethiopia is hardly hit by khat consumption. Both institution and community based studies done in Jimma have indicated the high prevalence of khat use seeing that 46 and 37.8%, respectively, chewed khat at least once in their lifetime [7, 8]. Previously, khat was mainly cultivated and chewed in limited, particularly eastern, part of the country. Nowadays, however, it is grown and consumed in all parts of the country including Amhara region [2, 9].

The recent sharp increment in khat consumption brings socio-economic, psychological and physical health sequelae of individuals involved. Though chewing khat is reported to induce a state of elation and feelings of increased alertness and arousal, at the end of a khat session, users may experience a depressed mood, irritability, loss of appetite, gastritis and peptic ulcer disease and difficulty sleeping [3, 10].

In addition, different studies in Ethiopia have revealed that the habit of chewing khat leads to a fragment of family, and multiple sexual practices. The habit, thus, could fuel spread of sexually transmitted infections (STIs) due to the associated risky sexual behaviors like having casual sex, unprotected sex and early initiation of sexual activity reported among chewers [11–15].

The number of khat users has increased in Ethiopia during recent decades and the habit has become popular in all sections of the society. Although khat is a legal substance in the country, it can be an entry point to the use of other illicit drugs, and risky sexual practices. This has to be made aware to local governmental officials and other concerned bodies to reduce its consequences. However, despite the prevalence of khat use and its effects were studied at institution basis, still, there is limited knowledge on khat chewing and its relation with risky sexual behaviors at the community level. Therefore, it is imperative to do this research determining the prevalence of lifetime and current chewing khat and its relation to risky sexual behaviors among residents of Bahir Dar City administration, Northwest Ethiopia. The findings will assist health care providers and program planners focusing their approach in the management of health problems related to khat consumption.

## Methodology

### Study design and period

A community based cross-sectional study was conducted to assess the level of chewing khat and its relation to risky sexual behaviors among residents of Bahir Dar city administration, Northwest Ethiopia from February to March 2016.

### Study area and population

The study was conducted in Bahir Dar city administration which is the capital city of Amhara national regional state. The city is located in Northwest Ethiopia around 565 km from Addis Ababa, the capital city of Ethiopia. The city is divided into 9 sub cities and 17 kebeles, and Tisabay, Zege, and Meshenti kebeles are currently under the city administration. In the city and around it, most of the farmers substitute their crop farm with khat cultivation to increase their income [16]. This availability of substance may precipitate community to use khat for people who live in the city and out of the city throughout the country. All populations aged 18 years and above who were invited for interview from selected kebeles of Bahir Dar City administration were study population.

### Inclusion and exclusion criteria

All people aged 18 years and above who lived for at least 6 months in selected kebeles during the study period and volunteer was included for the interview. However, relatives who came and family members who were not present during the study period were excluded. Those who were unable to communicate and seriously ill were excluded from the interview.

### Sample size determination

The sample size was determined using a formula for estimating a single population proportion. Considering 95% confidence interval (CI) (Zα/2 = 1.96), 5% margin of error and 37.8% proportion of khat chewing taken from a previous study [8] generated a minimum sample size of 361. After adjusting with 10% as non-response rate and two as design effect, a total of 794 respondents were recruited in the study.

### Sampling technique and procedure

The representative sample size was selected using multistage sampling technique. Ten kebeles out of total 20 were selected randomly using lottery method. Certain villages from each kebele were randomly selected. Therefore, a total of 794 households were distributed proportionally to the size of households in the selected kebeles, and villages based on a number of houses in selected kebeles and villages, respectively, referred from recent kebele registration. The shared households for each village were divided by a total number of households in a given village to determine a sampling interval and then households were selected by systematic random sampling technique. One individual was selected randomly from members of the household aged 18 years and above.

### Data collection and quality control

The pretested interviewer administered structured questionnaire was used to collect the data. The designed questionnaire was initially developed in English and translated first into the local language (Amharic) and then back to English with expertise to ensure its consistency. The questionnaire was pretested on 5% of total sample size in one non-selected Kebele, and modification and correction were done 1 week before data collection. The respondents were interviewed in their own language (Amharic). Three data collectors (BSc Nurse) and one supervisor (experienced public health officer) were selected and 2-day training was given to orient them on the tools to be used, the purpose of the study and how to approach respondents and obtain consent. The filled questionnaires were checked for completeness and consistency by supervisors. Incomplete questionnaires were returned to data collectors on the following day for the correction by revisiting the households.

The operational definitions used in this study were (a) lifetime chewer: an individual is considered an ever chewer even if he/she had chewed only once in his/her lifetime; (b) current user: individuals who were chewing khat within 3 months preceding the study; (c) having extra sexual partner: an individual is considered as having extra sexual partner if ever married individual had sexual partner in addition to his/her spouse; (d) ever had sexual intercourse: an individual is considered as an ever had sexual intercourse if a single individual had history of having sexual intercourse even only once in his/her lifetime; (e) inconsistent condom use: rarely, never and occasionally use of condoms during sexual contact other than always; (f) sexual behavior: an individual is considered as having risky sexual behavior if he/she reported having penetrative vaginal sex without using condom or inconsistently use with any partner rather than regular partner, having multiple sexual partners, having extra sexual partners, starting sexual intercourse before 18 years, watching pornographic film and sex with causal or commercial sex worker. In this study, respondents who engaged in at least one of the above behavior were considered as having risky sexual behavior.

### Data processing and analysis

The data was entered into computers using Epi-data version 3.1 and exported to SPSS version 20 for analysis. Percentage, frequency, and mean were used to describe the study participants in relation to relevant variables using tables. Chi-square (× 2) and/or Fisher’s exact test were applied to assess associations between variables. Logistic regression was performed to assess the association between outcomes and different explanatory variables. The strength of association was interpreted using odds ratio and 95% confidence interval. *P* value < 0.05 was considered statistically significant in this study.

## Results

### Socio-demographic characteristics of the study participants

All of the recruited study participants were interviewed yielding response rate of 100%; off them, 99.6% were urban dwelling and the remaining were from rural area. Majorities 457 (57.6%) of study participants were male and 408 (51.4%) were married. Of the total study participants, 249 (39.3%) were in age group of 25–34 years old, 253 (31.9%) grade 9–12 by education, and 384 (48.4%) had an average monthly income of ≤ 1000 Ethiopian Birr. Seven hundred eighty-three (98.6%) participants were Amhara by ethnicity and 623 (78.5%) were orthodox Tewahedo followers (Table 1).

**Table 1:** Socio-demographic characteristics of study participants, Bahir Dar City administration, Northwest Ethiopia, 2017

Variables	Category	Frequency (%)
Sex	Male	457 (57.6)
	Female	337 (42.4)
Age	18–24	249 (31.4)
	25–34	312 (39.3)
	35–44	114 (14.4)
	≥ 45	119 (15.0)
Residence (Sub city)	Abay Mado	182 (22.9)
	Belay Zeleke	222 (28.0)
	Fasilo	129 (16.2)
	Meshenti (satellite kebele)	25 (3.1)
	Shum Abo	163 (20.5)
	Zenzelima (satellite kebele)	73 (9.2)
Occupation	Government employ	213 (26.8)
	Merchant	254 (32.0)
	Daily laborer	126 (15.9)
	Student	96 (12.1)
	Farmer/housewife/private	105 (13.2)
Education	No formal education	165 (20.8)
	1–8 grade	134 (16.9)
	9–12 grade	253 (31.9)
	Diploma and above	242 (30.5)
Religion	Orthodox Tewahedo	623 (78.5)
	Muslim	147 (18.5)
	Others	24 (3)
Ethnicity	Amhara	783 (98.6)
	Others	11 (1.4)
Marital status	Married	408 (51.4)
	Single	351 (44.2)
	Widowed/separated	35 (4.4)
Average monthly income	< 1000 EBR	384 (48.4)
	1001–2000 EBR	223 (28.1)
	2001–3000 EBR	102 (12.8)
	≥ 3001 EBR	85 (10.7)

### Prevalence of khat chewing

Of the total 794 study participants, 204 (25.7%) had a history of chewing khat at least once in their life; of them, 79.9% had the history of chewing in the last 12 months. About 95 percent of those who chewed in the last 12 months practiced khat chewing within last 3 months. When the total 794 study participants were considered, 163 (20.5%) and 155 (19.5%) of participants chewed khat within last twelve and 3 months, respectively. Among 155 study participants who were practicing khat chewing currently (within last 3 months), 68 (43.9%) chewed weekly followed by 66 (42.6%) daily, 16 (10.3%) occasionally and 5 (3.2%) monthly (Data not shown in table).

### Reasons for practicing khat chewing

The study participants stated a number of ideas to be considered as reasons for practicing khat chewing. These included fighting depressed mood and getting concentration were mentioned by 56.9 and 45.6% of study participants, respectively (Table 2).

**Table 2:** Reasons stated by study participants for practicing khat chewing, Bahir Dar City administration, Northwest Ethiopia, 2017

Variables	Category	Khat chewing (%)
To get concentration	Yes	93 (45.6)
	No	111 (54.4)
Relieving emotional problems	Yes	57 (27.9)
	No	147 (72.1)
Desiring experiment	Yes	43 (21.1)
	No	161 (78.9)
Easily availability of khat	Yes	29 (14.2)
	No	175 (85.8)
Fighting depressed mood	Yes	116 (56.9)
	No	88 (43.1)
Religious purpose	Yes	78 (38.2)
	No	126 (61.8)
Availability of excess pocket money	Yes	7 (3.4)
	No	197 (96.6)
Socialization	Yes	52 (25.5)
	No	152 (74.5)
Getting acceptance by others	Yes	27 (13.2)
	No	177 (86.8)
Passing time	Yes	65 (31.9)
	No	139 (68.1)
Getting personal pleasure	Yes	81 (39.7)
	No	123 (60.3)
Peer pressure	Yes	69 (33.8)
	No	135 (66.2)
Lack of job	Yes	27 (13.2)
	No	177 (86.8)

### Problems encountered on khat chewers

The study participants mentioned several problems which are occurred due to their khat chewing practice. The participants thought that they lost money to get khat as stated by 67 (32.8%) of the total 204 chewers. Khat chewing also influenced their social relationships with their friends and parents as mentioned by 45 (22.1%) of chewers. Moreover, they were also exposed to physical health problems like injury and engagement in unprotected sex (Table 3).

**Table 3:** Problems encountered by study participants due to khat chewing, Bahir Dar City administration, Northwest Ethiopia, 2017

Variables	Category	Khat chewing (%)
Accident or injury	Yes	13 (6.4)
	No	191 (93.6)
Losses money	Yes	67 (32.8)
	No	137 (67.2)
Damage to objects or clothing	Yes	13 (6.4)
	No	191 (93.6)
Problem on relation with families	Yes	45 (22.1)
	No	159 (77.9)
Problem on relation with friends	Yes	18 (8.8)
	No	186 (91.2)
Problem on relation with others	Yes	12 (5.9)
	No	192 (94.1)
Poor performance at work	Yes	19 (9.3)
	No	185 (90.7)
Victim by robbery or theft	Yes	11 (5.4)
	No	193 (94.6)
Trouble with police	Yes	11 (5.4)
	No	193 (94.6)
Hospitalized at emergency	Yes	8 (3.9)
	No	196 (96.1)
Engaged in sex they regretted next day	Yes	12 (5.9)
	No	192 (94.1)
Engaged in unprotected sex	Yes	13 (6.4)
	No	191 (93.6)
Blackouts or flashbacks	Yes	15 (7.4)
	No	189 (92.6)
Other medical problems	Yes	11 (5.4)
	No	193 (94.6)
Quarrel/fight/scuffle	Yes	43 (21.1)
	No	161 (78.9)

### Risky sexual behaviors

Of the total 443 married/divorced/separated/widowed participants, 44 (9.93%) had extra sexual partners. Fifteen (34.1%) of those who had extra sexual partners had more than one extra sexual partners. Near to 56 percent of the total 351 single study participants had a history of sexual intercourse at least once in their life. Of these, 161 (82.1%) had experienced sexual intercourse within the last 12 months before the study. Out of the total 195 study participants who had a history of sexual intercourse at least once in life, 95 (48.7%) experienced sexual intercourse with causal partners and/or commercial sex workers and the remaining 100 (51.3%) practiced with regular partners.

In addition, 113 (70.8%) of 161 participants who had experienced sexual intercourse used a condom during their sexual activity with a condition of consistent (59.3%) and inconsistent (40.7%) usage. Regarding their self-reported sexually transmitted infections (STIs), 68 (8.6%) of the total participants had the history of STIs within last 12 months before this study. Moreover, 2.5% the total participants watched pornographic film (data not shown in table).

### Determinant factors associated with the prevalence of khat chewing

Results from the bivariate analysis have shown that sex, age, marital status, educational level, occupational status and average monthly income had statistically significant association with the prevalence of khat chewing. All these six variables were considered for the multiple logistic regression analysis. Except for educational status, the remaining five variables were retained as significantly associated factors of khat chewing among participants. The results confirmed that males had five times higher odds for chewing khat compared to females (AOR 5.0; 95% CI 3.0–8.2). Similarly, participants aged 25–34 years had 2.4 times higher odds for khat chewing compared to participants aged 18–24 years (AOR 2.4; 95% CI 1.4–4.1). Moreover, merchant study participants were near to five times more likely to chew khat than government employs (AOR 4.9; 95% CI 2.6–9.3). Likewise, those study participants who had more than three thousands average monthly income were 2.4 times more likely to chew khat than those who had less than one thousand average monthly income (AOR 2.4; 95% CI 1.2–4.8) (Table 4).

**Table 4:** Determinant factors associated with the prevalence of khat chewing among study participants, Bahir Dar City administration, Northwest Ethiopia, 2017

Variables	Category	Khat chewing		COR (95%CI)	AOR (95%CI)
		No	Yes		
Sex	Male	284 (62.1)	173 (37.9)	*6.0 (4.–9.1)*	*6.2 (4–9.7)*
	Female	306 (90.8)	31 (9.2)	1.00	1.00
Age	18–24	196 (78.7)	53 (21.3)	1.00	1.00
	25–34	215 (68.9)	97 (31.1)	*1.7 (1.1–2.5)*	*2.1 (1.3–3.5)*
	35–44	90 (78.9)	24 (21.1)	1.0 (0.6–1.7)	1.7 (0.8–3.4)
	≥ 45	89 (74.8)	30 (25.2)	1.3 (0.8–2.1)	*2.0 (1.0–4.3)*
Marital status	Married	313 (76.7)	95 (23.3)	1.00	1.00
	Single	247 (70.4)	104 (29.6)	*1.4 (1.0–1.9)*	*1.7 (1.1–2.7)*
	Widowed/separated	30 (85.7)	5 (14.3)	0.6 (0.2–1.5)	1.0 (0.3–2.9)
Education	No formal education	132 (80.0)	33 (20)	1.00	1.00
	1–8 grade	90 (67.2)	44 (32.8)	*2.0 (1.2–3.3)*	*1.8 (1.0–3.4)*
	9–12 grade	180 (71.1)	73 (28.9)	*1.6 (1.0–2.6)*	1.3 (0.7–2.5)
	Diploma and above	188 (77.7)	54 (22.3)	1.2 (0.7–1.9)	1.0 (0.5–2.1)
Occupation	Government employ	177 (83.1)	36 (16.9)	1.00	1.00
	Merchant	162 (63.8)	92 (36.2)	*2.8 (1.8–4.3)*	*3.3 (1.9–5.9)*
	Daily laborer	101 (80.2)	25 (19.8)	1.2 (0.7–2.1)	1.9 (0.9–4.1)
	Student	74 (77.1)	22 (22.9)	1.5 (0.8–2.7)	*3.3 (1.4–7.5)*
	Farmer/housewife/private	76 (72.4)	29 (27.6)	*1.9 (1.1–3.3)*	*4.3 (2.0–9.1)*
Average monthly income	≤ 1000 EBR	308 (80.2)	76 (19.8)	1.00	1.00
	1001–2000 EBR	160 (71.7)	63 (28.3)	*1.6 (1.1–2.3)*	*1.8 (1.1–2.9)*
	2001–3000 EBR	68 (66.7)	34 (33.3)	*2.0 (1.3–3.3)*	*2.3 (1.3–4.3)*
	≥ 3001 EBR	54 (63.5)	31 (36.5)	*2.3 (1.4–3.9)*	*2.2 (1.2–4.3)*

NB: Italic indicates significant value

*COR* crude odds ratio; *AOR* adjusted odds ratio; *95%CI* 95% confidence interval

### Risky sexual behaviors and their relation to khat chewing practice

The association of at least once in life khat chewing and alcohol drinking practice with risky sexual behaviors were analyzed using bivariate logistic regression. And then, those variables that fulfilled P-value < 0.2 were entered into multiple logistic regressions for further analysis. Furthermore, the association of those variables with less than five observation was analyzed using only cross-tabulation and Fisher’s Exact Test. Table 5 has shown the relationships of khat chewing practice with some risky sexual behaviors suggesting that khat chewing has significantly increased the odds of having extra sexual partners for married (AOR: 2.2; 95% CI 1.1–4.6); ever had sexual intercourse for single (AOR 2.5; 95% CI 1.1–4.3); pornographic film watching(AOR 4.0; 95% CI 1.3–12.8) and self-reported STIs (AOR 8.3; 95% CI 4.6–15.1) by 2.2, 2.5,4.0 and 8.3 times, respectively, when compared to those not chewing khat.

**Table 5:** Relation of khat chewing with risky sexual behaviors among study participants, Bahir Dar City administration, Northwest Ethiopia, 2017

Variables	Category	Having extra sexual partner (married)	ever had sexual intercourse (single)	Having sexual intercourse within last 12 months (single)	sexual partners	No condom use	Inconsistent condom use	Pornographic film watching	Self-reported STIs
Sex	Male	*2.0 (1.0–3.8)*	0.8 (0.5–1.3)	1.5 (0.7–3.1)	*2.1 (1.2–3.8)*		0.6 (0.3–1.2)	0.25**	*2.8 (1.6–5.1)*
	Female	1.00	1.00	1.00	1.00	1.00	1.00		1.00
Age	18–24	1.00	1.00	1.00	1.00	1.00	1.00	0.37**	1.00
	25–34	1.0 (0.4–2.5)	*3.2 (2.0–5.1)*	1.6 (0.8–3.5)	1.1 (0.6–2.0)	0.8 (0.4–1.6)	1.1 (0.6–2.3)		1.7 (0.9–3.1)
	>35	0.8 (0.3–2.1)	*4.4 (1.7–11.5)*	1.2 (0.4–3.8)	*4.1 (1.5–11.2)*	0.6 (0.2–1.4)	0.6 (0.2–2.0)		1.4 (0.7–2.7)
Chewing khat	Yes	*2.2 (1.1–4.2)*	*2.0 (1.2–3.2)*	1.5 (0.7–3.1)	1.2 (0.7–2.0)	0.9 (0.5–1.6)	1.1 (0.6–2.2)	*3.9 (1.3–12.3)*	*8.1 (4.7–14.0)*
	No	1.00	1.00	1.00	1.00	1.00	1.00		1.00
Alcohol use	Yes	*2.1 (1.1–3.9)*	*3.0 (1.9–4.6)*	1.5 (0.8–3.0)	1.2 (0.7–2.0)	1.7 (0.9–3.0)	1.1 (0.5–2.1)	*4.4 (1.2–15.5)*	*2.2 (1.3–3.7)*
	No	1.00	1.00	1.00	1.00	1.00	1.00		1.00
Sex*	Male	*1.4 (0.7–2.9)*	0.4 (0.2–0.7)	NA	*2.4 (1.2–4.8)*	1.6 (0.8–3.0)	NA	NA	1.2 (0.6–2.3)
	Female	1.00	1.00		1.00	1.00			1.00
Age*	18–24	1.00	1.00		1.00	1.00			1.00
	25–34	0.7 (0.3–2.0)	*3.1 (1.9–5.0)*	NA	1.0 (0.6–1.9)	0.8 (0.4–1.5)	NA	NA	1.2 (0.6–2.4)
	>35	0.7 (0.3–1.9)	*3.4 (1.3–9.3)*		*4.0 (1.4–11.2)*	0.5 (0.2–1.3)			1.5 (0.7–3.1)
Chewing khat*	Yes	*2.2 (1.1–4.6)*	*2.5 (1.4–4.3)*	NA	0.8 (0.4–1.6)	NA	NA	*4.0 (1.3–12.8)*	*8.3 (4.6–15.1)*
	No	1.00	1.00		1.00				1.00
Alcohol use*	Yes	*2.1 (1.1–4.2)*	*3.2 (2.0–5.3)*	NA	0.9 (0.5–1.7)	1.5 (0.8–2.9)	NA	*4.5 (1.2–16.1)*	*2.6 (1.4–4.5)*
	No	1.00	1.00		1.00	1.00			1.00

NB: *Italic* indicates significant value

*NA* not applicable

* Adjusted OR (95% CI)

** Fisher’s Exact Test

Alcohol drinkers had an increased odds of having extra sexual partners (AOR 2.1; 95% CI 1.1–4.2); ever had sexual intercourse (AOR 3.2; 95% CI 2.0–5.3); watching pornographic film (AOR 4.5; 95% CI 1.2–16.1), and self-reported STIs (AOR 2.6; 95% CI 1.4–4.5) by more than two times when compared to those who did not drink (Table 5).

## Discussion

The increment in the number of khat chewers globally needs a better understanding of the local burden and most common influencing factors and its relation to risky sexual behaviors. This paper has found that life time and current prevalence of chewing khat were 25.7 and 19.5%, respectively. The life time prevalence of the current study is in line with the findings in Eastern Ethiopia (24.2%) [17] and Ethiopian University students (24%) [18]. Nevertheless, this study has revealed relatively higher lifetime and current chewing khat prevalence than the result from Jimma, Ataye, Lalibela, Gondar and Dera parts of Ethiopia (14.3–17.9% and 13.3–14.2%, respectively) [19–22].

The higher prevalence of khat chewing in the current study and Eastern Ethiopia might be due to the fact that these two sites cultivate the substance leading availability of khat with least cost. Easily accessibility of substance and its low cost exposes the community to develop habits of chewing [16, 18, 23].

The lifetime (42.0%) and current (32.5%) prevalence of chewing khat in Gondar town Northwest Ethiopia [24] were higher than 25.7 and 19.5%, respectively, from the current study. The difference might be due to the difference in the study participants. The study done in Gondar consisted only college students, but the current study included both students and other participants in the community. According to the similar study, students who perceived that khat helps to study better were more likely to chew khat than those who did not perceive [18]. This has shown that students were expected to use khat to enhance their academic performance which is kept in a report from Kenya [25]. This is also supported by the current study indicating that being students increased the habit of chewing khat compared to government employees.

In this study, the most commonly mentioned reasons for chewing khat are fighting depressed mood (56.9%) followed by getting concentration (45.6%). Similar findings were also documented from the study done in Gondar town northwest Ethiopia where concentration during the study and getting entertainment and relaxation were reported by 62.3 and 36.9% of participants, respectively [24]. In addition, religious purpose, peer pressure, passing time and socialization were also mentioned as reasons of chewing khat in the current and previous studies done in University of Gondar and Dera district [9, 18].

The present study showed that males had around six times more probability of chewing khat than females which is consistent with studies in Ethiopia and Saudi Arabia [9, 17, 20, 22, 26, 27]. This might be due to the fact that the number of substance users is high among males than females, and it is accepted to use substances among males in different parts of the world including Ethiopia. In other words, this could be due to the culture which discourages the habit of chewing khat among females. This is also supported by the study done in Kenya showing that respondents strongly agreed on luxuriated habit of Khat chewing more boys than girls [25].

Likewise, occupation, marital status, and average monthly income were the most important parameters either to increase or hinder the chance of chewing khat in this study which is in line with other study done elsewhere [28]. Merchants and students practiced the habit near to five times more than government employees. The students might use the substance for getting concentration during the study and due to peer pressure since students pass their time with their friends. Students used chewing khat to study better than not used [18]. Having friends with khat chewing and peer pressure were confirmed as they scupper the people for a habit of chewing. More than half of respondents under the influence of their peer used substances [29]. The current study has also revealed that merchants were more likely to chew khat than government employees which is in line with the report from Dera district, Ethiopia [9].

The study participants with higher incomes in this study were more likely for chewing khat than to those with relatively lower incomes. This thought has concurred with the study done in Yemen [30] and University students of Ethiopia [17]. Furthermore, study participants with highest family socio-economic status were more prone to substance use [22]. This might be due to lack/shortage of money to buy khat since diversion of income for purchase of khat results in neglecting the family needs leading to family conflicts and discords. Thus, participants with low income will be restricted from chewing khat.

Khat chewing brings socio-economic and physical health problem on chewers. The current study participants mentioned that they lost their money performed their work poorly and damaged their objects to get khat which is in coherent with results reported from northwest Ethiopia [31, 32]. Moreover, khat chewing influenced their social relationships with their friends, parents, and others. This might be due to the reason that they lost their time on chewing khat resulting in a shortage of time to participate in any social related activities. For instance, chewers lost an average of three and half hours for single chewing practice without considering the time lost for buying and preparation according to this study. In addition, this study found that the chewers’ physical health including exposure to STIs is also damaged due to their practice.

This study has revealed that chewing khat has a significant association to increase odds of developing risky sexual behaviors which are endorsed by other study done in Dilla University, Ethiopia [33]. Studies were done in the United Kingdom and Ghana have shown the increased impact of substance use on taking of risky sexual behaviors though they assessed the effect of other substances rather than khat [26, 34]. These risky sexual behaviors included having extramarital sexual partners; ever had sexual intercourse; and self-reported STIs. The significant relation of initiation of sexual intercourse and chewing khat was similarly reported in Dilla University, Ethiopia [33]. Moreover, those substance users were actively engaged with multiple sexual partners according to the study done in Ghana among homeless children and adolescents [26]. Chewing khat and alcohol drinking habit could also place the married individuals to have extramarital sexual relationships according to the present result which may facilitate the transmission of STIs. Similar findings reported from Bahir Dar University, Ethiopia resulting in having multiple sexual partners and sex with commercial sex workers, and sexual intercourse for money generation [35].

The self-reported STIs might be due to their inconsistent use of condoms during their sexual activity which was documented in the current study reporting that near to six percent of the participants engaged in unprotected sex. Similarly, another study conducted in Ghana [26] is in line with the present study. Although the study was not done on khat chewers, the finding from Sao Paulo Brazil has supported the effect of substance use on inconsistent utilization of condom [36]. The current study also documented not using of condom among alcohol drinkers during their last sexual activity that may increase the prevalence of self-reported STIs since alcohol drinking increased risky sexual behavior as affirmed by a study in Pawe District and Bahir Dar University, Ethiopia [35, 37]. It is not statistically significant; however, khat chewers have shown slightly increased odds of having sexual intercourse within last 12 months and inconsistent use of a condom.

In general, the discussion, implication, and conclusion of this study were performed by considering the limitation of the study. The relation of khat chewing with risky sexual behaviors was not possible to make clear cause-effect relationship since the study design was cross-sectional. In addition, the data were collected using questionnaires adopted and adapted by reviewing different published articles indicating not assessed by a standard tool which was another limitation. Psychological morbidity was not screened suggesting that the paper did not address the effect of chewing khat on psychological health.

## Conclusion

Khat chewing is relatively prevalent among community residents in the study area which is significantly associated with male gender, being a merchant, student and having an average of more than 1001 EBR monthly income. This study also examined that khat use was found to be a risk factor for the development of risky sexual behaviors exposing them at risk for acquisition and transmission of STIs. Therefore, creating community awareness to address the problem of khat chewing and related consequences was recommended to health professionals and volunteer community health workers. The result demands an integrated strategy to effectively control both khat use and related STIs. In addition, further studies are needed using longitudinal study design to explore the actual interactions between khat use and risky sexual behaviors among community residents.
